# A Nondestructive Eggshell Thickness Measurement Technique Using Terahertz Waves

**DOI:** 10.1038/s41598-020-57774-5

**Published:** 2020-01-23

**Authors:** Alin Khaliduzzaman, Keiji Konagaya, Tetsuhito Suzuki, Ayuko Kashimori, Naoshi Kondo, Yuichi Ogawa

**Affiliations:** 1 Laboratory of Bio-Sensing Engineering, Graduate School of Agriculture, Kyoto University, Kyoto, 606-8502 Japan; 2 Research and Development Division, Nabel Co., Ltd., Kyoto, 601-8444 Japan; 3 Faculty of Agricultural Engineering and Technology, Sylhet Agricultural University, Sylhet, 3100 Bangladesh; 4 JSPS International Research Fellow, Graduate School of Agriculture, Kyoto University, Kyoto, 606–8502 Japan

**Keywords:** Ecology, Optical spectroscopy, Animal physiology

## Abstract

Eggshells play a number of important roles in the avian and reptile kingdom: protection of internal contents and as a major source of minerals for developing embryos. However, when researching these respective roles, eggshell thickness measurement remains a bottleneck due to the lack of a non-destructive measurement techniques. As a result, many avian and reptile research protocols omit consideration of eggshell thickness bias on egg or embryo growth and development. Here, we validate a non-destructive method to estimate eggshell thickness based on terahertz (THz) reflectance spectroscopy using chicken white coloured eggs. Since terahertz waves are reflected from outer air-eggshell interface, as well as the inner eggshell-membrane boundary, the resulting interference signals depend on eggshell thickness. Thus, it is possible to estimate shell thickness from the oscillation distance in frequency-domain. A linear regression-based prediction model for non-destructive eggshell thickness measurement was developed, which had a coefficient of determination (R^2^) of 0.93, RMSEP of 0.009, RPD of 3.45 and RER 13.67. This model can estimate eggshell thickness to a resolution of less than 10 μm. This method has the potential to expand the protocols in the field of avian and reptile research, as well as be applied to industrial grading of eggs.

## Introduction

The eggshell is an amazingly efficient natural package for protecting nascent biological systems. This includes protection of internal contents from contaminants and embryos from UV radiation^1^, as well as facilitating respiratory gas exchange^2^. It is also a valuable source of minerals (e.g. calcium) for embryo development of all birds and reptiles^3,4^. To date, however, this eggshell has also been a physical barrier for non-invasive *in-vivo* research. In particular, a lack of non-destructive eggshell thickness measurements has constrained avian and reptile egg and embryo research protocols in various scientific fields (e.g. ornithology, developmental animal biology, physiology, ecology, bio-sensing engineering, food engineering, veterinary science, zoology, animal husbandry).

For instance, the optical or acoustic signals used to measure avian and reptile eggs in numerous studies (e.g. spectroscopic measurement) can be influenced by shell thickness^5,6^. Moreover, thicker eggshells (i.e. higher deposited resources) could provide extra benefit (i.e. higher protection, nutrients and minerals) to the internal contents and/or embryos, since eggshell is a major source of minerals for embryonic development^4,7^. Thus, it is crucial that this potential bias in current scientific research methodologies be accounted for. Therefore, any technique that can measure eggshell thickness non-destructively will make a significant contribution to avian research. Electromagnetic waves in the UV, visible and even NIR regions are limited in their ability to probe eggshell properties due to high absorbance and scattering effects. Moreover, eggshell colour pigment (i.e. protoporphyrin) further masks measurements in the visible region^8^. Therefore, we examined the use of THz waves (electromagnetic waves with a lower frequency than the above) to measure chicken eggshell thickness. Terahertz waves are a relatively new, under-explored, part of the electromagnetic wave range that promises to be an extremely useful research tool for egg research. In particular, terahertz pulses are suited to examining components with a crystalline structure and low moisture content like avian eggshells^9^. In materials with a higher moisture content, the THz waves are strongly absorbed by bulk water^10,11^. The moisture content of the chicken eggshell is very low, less than 1.0%. Moreover, the main component of the eggshell, calcite, is calcium carbonate (CaCO_3_), which is a crystalline and asymmetric chemical compound^12^. Given this, the present research developed a non-destructive method to estimate eggshell thickness using a THz pulse.

## Results

**Figure 1:**
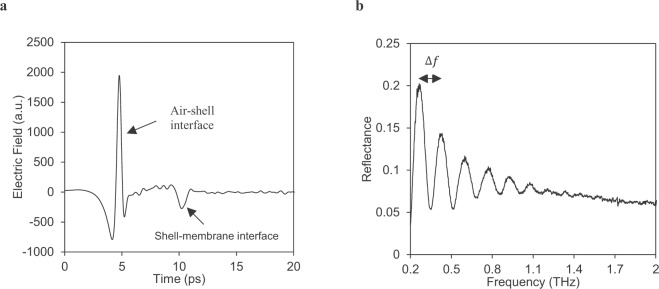
Reflected THz pulses of chicken egg using THz Time-domain spectroscopy (THz-TDS). (**a**) Reflected time-domain THz pulse signal from an intact eggshell. The peak in time-domain signal appeared at air-eggshell interface whereas second peak at shell-membrane interface. (**b**) Frequency-domain (interference) signal of a chicken intact eggshell. The interference signal was more prominent from 0.2 to 1.2 THz.

When a THz pulse is sent towards the egg there are two distinctive reflection peaks in the time-domain signal: the first from the air-outer eggshell and then from the inner shell-membrane interface (Fig. 1a). The frequency-domain THz signal of intact chicken eggs oscillated from 0.204 to 1.203 THz (Fig. 1b). This oscillation is dependent on eggshell thickness, chemical compounds and the crystalline structure of the eggshell. Terahertz interference of eggshell may be involved in amplitude differences at various frequency in the 0.2 to 2.0 THz due to the differences in transmittance of the various wave bands. The larger the peak to peak distance, the thinner the eggshell that means the distance (∆*f)* is inversely proportional to eggshell thickness.

The eggshell thickness of white coloured eggs (layer breed) was estimated by the following Eq. 1. This equation was developed based on a linear regression of the inverse of peak to peak distance in frequency-domain signal (1/∆*f*) and actual eggshell thickness (*T*_a_) for the training set. Equation 2 was derived from Eq. 1.

1 $$1/\Delta f=a\ast {T}_{{\rm{a}}}+b$$

2 $$1/\Delta f=22.547\ast {T}_{e}+1.8288$$

where, *T*_e_ is estimated eggshell thickness in millimeters for white shell coloured eggs, ∆*f* is the peak to peak distance of THz frequency-domain interference signal of the intact egg in reflection mode and *a*, *b* are the coefficients of the model where *a* is 22.547 and *b* is 1.8288 for white coloured eggshells. Coefficients may vary with the breed of the chicken. For example, different coefficient value based on the colour of the eggshell may be required. We may define same coefficient for all white coloured eggshell type and different coefficient for brown coloured eggs, where *a* is 19.125 and *b* is 0.4421 for brown coloured eggshells (Suppl. Fig. S1). Although a single model for all eggs could be used, separate models for white and brown eggs can work well to achieve better results.

**Figure 2:**
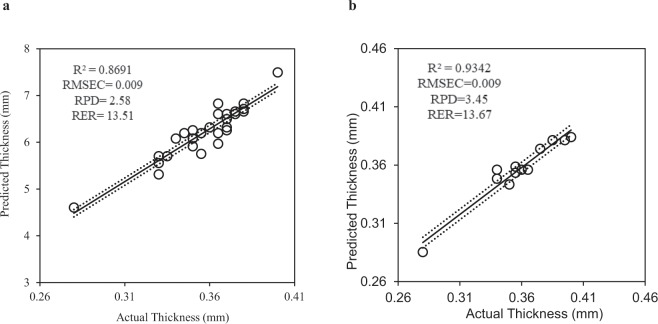
Chicken eggshell thickness measurement based on linear regression between inverse of peak to peak distance (1/∆*f*) and actual eggshell thickness (mm). (**a**) Training dataset for model development (dotted lines represent the 95% upper and lower confidence interval). (**b**) Testing dataset with 95% confidence interval lines (upper and lower).

The performance of this prediction model was evaluated using multiple parameters: coefficient of determination (R^2^), root mean square error of prediction (RMSEP), ratio of prediction difference (RPD) and range error ratio (RER) values. The estimated eggshell thickness was significantly correlated with actual eggshell thickness (R^2^ = 0.9342, RMSEP = 0.009). With a RMSEP of 0.009 mm, the model can estimate eggshell thickness to less than 10 μm resolution. The RPD and RER values of the model were 3.45 and 13.67 respectively, indicating excellent prediction performances (Fig. 2).

## Discussion

Three phenomena, reflection, transmission and attenuation processes, are important to consider during the propagation of THz waves at every interface (i.e. air-shell, shell-membrane and membrane-albumen interface) of an intact egg. Eggshell has a crystalline structure of CaCO_3,_ called palisades and mamillary cones^12^. The inside of the eggshell is covered by a separate soft membrane, called the shell membrane, composed of fibril protein followed by a thin albumen layer. The dry materials have low attenuation (due to low absorbance) of the terahertz pulse. In other words, most attenuation occurs according to the amount of water in the medium. The transmission and reflected pulses intensity depend both on refractive index (*n*) and extinction coefficient (*k*) of the material. In the terahertz region the refractive index depends on how compact the material (e.g. crystallinity, density) is and the moisture content of the material. The higher the refractive index is, the higher the reflection and lower the speed of the light will be when passing through the medium. The refractive index of eggshell with membrane was ~2.7 at 0.5 THz which indicated that the reflected THz pulses were greater at air-outer shell interface (Suppl. Fig. S2). On the other hand, a higher extinction coefficient means a higher light attenuation when passing through the medium. The extinction coefficient of the eggshell (~0.05 at 0.5 THz) was much lower than of the egg albumen (~0.76 at 0.5 THz), which indicates that the transmitted part of the terahertz pulse was largely absorbed by the egg white and attenuated soon (Suppl. Figs. S2 and S3). Moreover, the intensity of the incident THz pulse become decreased with increasing depth from the surface, since part of the incident THz pulse was reflected and absorbed (or attenuated) by the mediums. Therefore, the reflected THz pulse intensity should be much stronger from the shell-membrane interface than from the membrane-albumen interface. A part of the THz waves was reflected from the albumen, but the transmitted part was quickly attenuated (i.e. penetration depth should be low in the albumen). Interestingly, variations in intensity (of different egg samples) only influence the amplitude of the signal, not the oscillation phase of the interference signal (oscillation is due to the crystalized eggshell structure); thus the peak to peak distance (∆*f*) of the interference signal was not influenced.

The refractive index of the shell membrane should also be high because of its collagen fibrils content and tendon-like polypeptide structures (collagen fibrils have a high refractive index). It is quite difficult for separate study of shell membrane with the available device (e.g. THz-TDS). Hence, to separate them using a time-domain waveform, it is necessary to use a laser with a shorter pulse width. In this paper, we have assumed that the thickness of the shell membrane is constant in order to estimate the approximate thickness of the eggshell. The reflected THz oscillation (interference signal) in the frequency-domain signal was due to the crystalline structure of eggshell (i.e. calcite), not due to the membrane or egg white (non-crystalline part). Therefore, the reflected THz pulse showed two characteristic peaks in the time-domain signal; the first from the air-outer eggshell and the second from the inner shell-membrane interfaces.

Given that the observed egg spectrum closely resembled pure water, we can infer the predominant role of water on the spectra. Since water bounded to the shell has no absorption in this THz range^10,13,14^, water from the underlying egg features (e.g. albumin) inevitably affect the observed spectra, especially its general shape; that is except for the oscillation distance (Δ*f*). A more precise picture of THz wave propagation can be drawn based on the optical properties of egg components, and it would help to improve the measurement accuracy of the eggshell in the future works.

The distance between two oscillatory peaks of the frequency-domain signal was inversely proportional to the eggshell thickness. Perhaps, the terahertz interference signal was also influenced by pigment (e.g. protoporphyrin IX) content deposited on the eggshell. The major pigment in eggshells of brown-egg laying hens is protoporphyrin IX, but traces of biliverdin and its zinc chelates are also present^15^. Protoporphyrin IX is an organic compound that belongs to a group of families of biologically active tetrapyrrole compounds, where the suffix refers to the position of the side chain^16^. The asymmetric nature of the chemical structure of this compound may interfere with precise measurement for shell thickness in brown eggs. Therefore, the correlation was higher for the white eggshell (R^2^ = 0.87, Fig. 2a) than for the brown eggshell (R^2^ = 0.75, Suppl. Fig. S1).

In conclusion, eggshells have been a bottle neck (i.e. physical barrier) to non-destructive research of avian and reptile eggs and embryos prior to and during the incubation stage. We have developed and validated a technique that can measure eggshell thickness non-invasively. In this technique the egg was irradiated with a THz pulse, with the resulting reflected oscillatory signal in the frequency-domain being used as an indirect *in-vivo* measure of intact eggshell thickness. The distance between the two oscillating interference signals was inversely related to eggshell thickness. This eggshell thickness measurement protocol has the potential to be applied to many scientific fields (e.g. avian and reptile biology, ecology, ornithology, developmental biology, evolutionary biology, industrial grading of eggs) that encompasses many bird and reptile species worldwide^17^. Moreover, this technique potentially enables the exploration of many undiscovered functions of the eggshell in the near future. For example, eggs with a thicker eggshell might be preferred by farmers and other stakeholders for their hatcheries and laboratory samples. This is because eggs with a thicker eggshell may produce healthier offspring, since it is a major source of minerals; and better protects of internal contents against UV radiation (sun/light exposure).

## Materials and Methods

**Figure 3:**
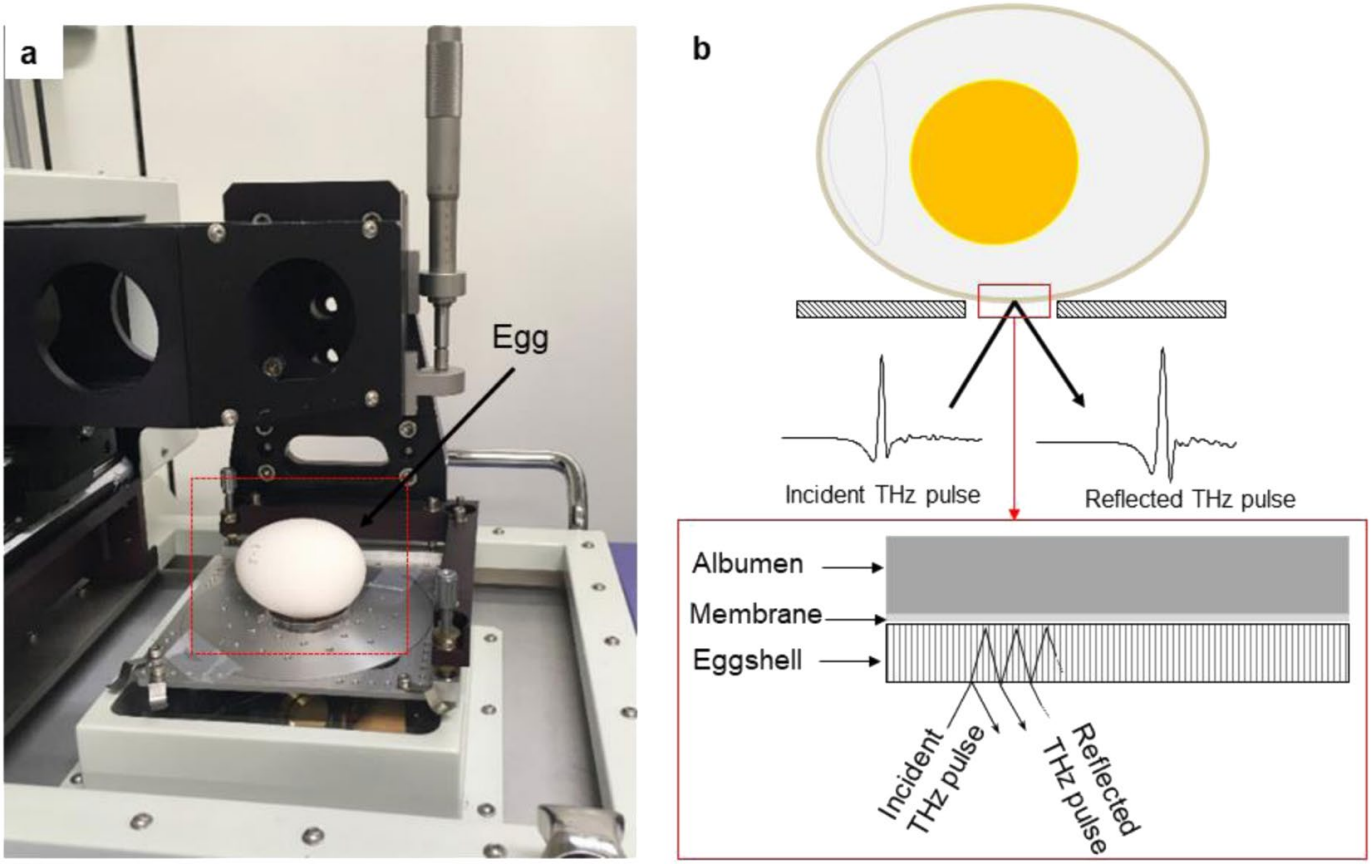
Signal (reflected THz pulses) acquisition of intact egg using THz-TDS. (**a**) Actual figure during signal acquisition using THz-TDS. Terahertz waves are electromagnetic waves with picosecond periodic vibrations. Femtosecond lasers were used in the THz-TDS to accurately measure the picosecond pulses (**b**) Schematic diagram of terahertz reflectance phenomena of intact eggshell. The signal was taken in horizontal position of egg to avoid air sac at upper end of egg and to minimize the degree of curvature of eggshell.

Infertile chicken market eggs (Brand: “FRESH EGG”) were used as a model sample for the experiments, which were conducted from 12^th^ to 27^th^ December 2018. The intact eggs were measured using terahertz time-domain spectroscopy (THz-TDS, model: TPS spectra 3000, TeraView Ltd., UK) shown in Fig. 3. The eggs were irradiated by a THz pulse with an incident angle of 13 degrees. Following this, the reflected time-domain signal from the egg was obtained and reflectance calculated using a Blackman-Harris window function^18^. We then performed a fast Fourier transformation (FFT). The spectra ranged in frequency from 0.2 to 2.0 THz with a resolution of 2 cm^−1^. The eggs were placed in a room overnight (12 h) at 24 ± 1 °C and ~50% RH prior to the THz measurement. The distance between the first two peaks (∆*f*) of frequency-domain signal was calculated automatically using an algorithm written in MATLAB 2016a (Mathworks, Natick, MA, USA) software. To develop a univariate regression model to indirectly estimate eggshell thickness, the actual shell thicknesses of all eggs were measured by a micrometer (Mitsutoyo Corp., Japan) after the THz measurement had been taken. Statistical analysis (e.g. univariate linear regression, 95% confidence interval lines, model development) was performed using Microsoft Excel (Microsoft Office 365) and MATLAB 2016a Software. The egg samples were divided into two groups i.e. a training set (70% data) and a testing set (30% data) for development and validation of the model. The performance of the prediction model was evaluated using coefficient of determination (R^2^), root mean square error of prediction (RMSEP), ratio of prediction difference (RPD) and range error ratio (RER) values. A good calibration model should have a high correlation coefficient (R) and a low RMSEP^8^. The RPD value, a measure of usefulness or goodness of fit, is higher when the error of prediction is smaller; thus higher RPD value means a better model^19^. An RPD between 1.5 and 2 indicates that the calibration model can distinguish between low and high values of the response variable; a value between 2 and 2.5 means that quantitative predictions are possible, and an RPD value between 2.5 and 3.0 indicates an excellent prediction performance. On the other hand, a RER value of 10 or higher indicates excellent performance of the model in actual application^20,21^.

## Supplementary information

Supplementary Information.

## Data Availability

The data that support the findings of this study are available from the corresponding author upon reasonable request. The codes used in this study are available from the corresponding author upon reasonable request.

## References

[1] Maurer G (2015). First light for avian embryos: Eggshell thickness and pigmentation mediate variation in development and UV exposure in wild bird eggs. Funct. Ecol..

[2] A.R A, Rahn H (1985). Pores in avian eggshells: Gas conductance, gas exchange and embryonic growth rate. Respir. Physiol..

[3] Richards MP (1997). Trace Mineral Metabolism in the Avian Embryo. Poult. Sci..

[4] Tuan RS, Ono T (1986). Regulation of extraembryonic calcium mobilization by the developing chick embryo. J Embryol Exp Morphol.

[5] Khaliduzzaman A (2019). Non-invasive broiler chick embryo sexing based on opacity value of incubated eggs. Comput. Electron. Agric..

[6] Khaliduzzaman A (2019). A non-invasive diagnosis technique of chick embryonic cardiac arrhythmia using near infrared light. Comput. Electron. Agric..

[7] Romanoff, A. L. & Romanoff, A. J. *Biochemistry of the Avian Embryo: A Quantitative Analysis of Prenatal Development*. (1967).

[8] Islam MH (2015). Prediction of chick hatching time using visible transmission spectroscopy combined with partial least squares regression. Eng. Agric. Environ. Food.

[9] Nakano T, Ikawa NI, Ozimek L (2003). Chemical composition of chicken eggshell and shell membranes. Poult. Sci..

[10] Xu J, Plaxco KW, Allen SJ (2006). Collective dynamics of lysozyme in water: Terahertz absorption spectroscopy and comparison with theory. J. Phys. Chem. B.

[11] Møller U, Cooke DG, Tanaka K, Jepsen PU (2009). Terahertz reflection spectroscopy of Debye relaxation in polar liquids [Invited]. J. Opt. Soc. Am. B.

[12] Hincke MT (2012). The eggshell: structure, composition and mineralization. Front. Biosci. (Landmark Ed..

[13] Bellissent-Funel MC (2016). Water Determines the Structure and Dynamics of Proteins. Chemical Reviews.

[14] Arikawa T, Nagai M, Tanaka K (2008). Characterizing hydration state in solution using terahertz time-domain attenuated total reflection spectroscopy. Chem. Phys. Lett..

[15] Samiullah S, Roberts JR, Chousalkar K (2015). Eggshell color in brown-egg laying hens-a review. Poultry Science.

[16] Sparks NHC (2011). Eggshell pigments - From formation to deposition. Avian Biology Research.

[17] Barrowclough GF, Cracraft J, Klicka J, Zink RM (2016). How many kinds of birds are there and why does it matter?. PLoS One.

[18] Vázquez-Cabo J (2016). Windowing of THz time-domain spectroscopy signals: A study based on lactose. Opt. Commun..

[19] Syduzzaman Md, Rahman Afzal, Alin Khaliduzzaman, Fujitani Shinichi, Kashimori Ayuko, Suzuki Tetsuhito, Ogawa Yuichi, Kondo Naoshi (2019). Noninvasive quantification of yolk content using Vis-NIR spectroscopy and its effect on hatching time and gender of broiler chicken. Engineering in Agriculture, Environment and Food.

[20] Rahman A (2015). Prediction of K value for fish flesh based on ultraviolet–visible spectroscopy of fish eye fluid using partial least squares regression. Comput. Electron. Agric..

[21] Suhandy D, Yulia M, Ogawa Y, Kondo N (2013). Prediction of L-Ascorbic Acid using FTIR-ATR Terahertz Spectroscopy Combined with Interval Partial Least Squares (iPLS) Regression. Eng. Agric. Environ. Food.

