# Supplementary material for: A Nondestructive Eggshell Thickness Measurement Technique Using Terahertz Waves
Source: Sci Rep. 2020 Jan 23;10:1052. doi: 10.1038/s41598-020-57774-5 (PMC6978418; doi:10.1038/s41598-020-57774-5)
Supplement: Supplementary file 1 — Supplementary Information. [file 41598_2020_57774_MOESM1_ESM.pdf]

Title

A Nondestructive Eggshell Thickness Measurement Technique Using Terahertz Waves

Authors

Alin Khaliduzzaman[1,3,4,*], Keiji Konagaya[1], Tetsuhito Suzuki[1], Ayuko Kashimori[1], Naoshi Kondo[1], Yuichi Ogawa[1,*]

Author information

[1]Laboratory of Bio-Sensing Engineering, Graduate School of Agriculture, Kyoto University, Kyoto 606-8502, Japan

[2]Research and Development Division, Nabel Co., Ltd., Kyoto 601-8444, Japan

[3]Faculty of Agricultural Engineering and Technology, Sylhet Agricultural University, Sylhet 3100, Bangladesh

[4]JSPS International Research Fellow, Graduate School of Agriculture, Kyoto University, Kyoto 606-8502, Japan

* Corresponding authors: correspondence should be addressed to khaliduzzaman.88s@st.kyoto-u.ac.jp or ogawayu@kais.kyoto-u.ac.jp

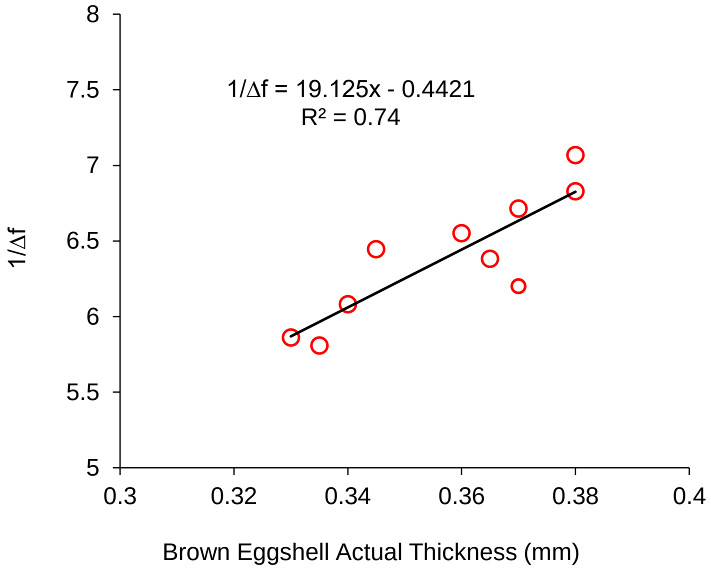

Suppl. Fig. S1. Relationship between actual thickness of brown eggshell and peak to peak distance of THz frequency domain interference signal. The performance of the model is influenced by pigment of eggshell called protoporphyrin IX

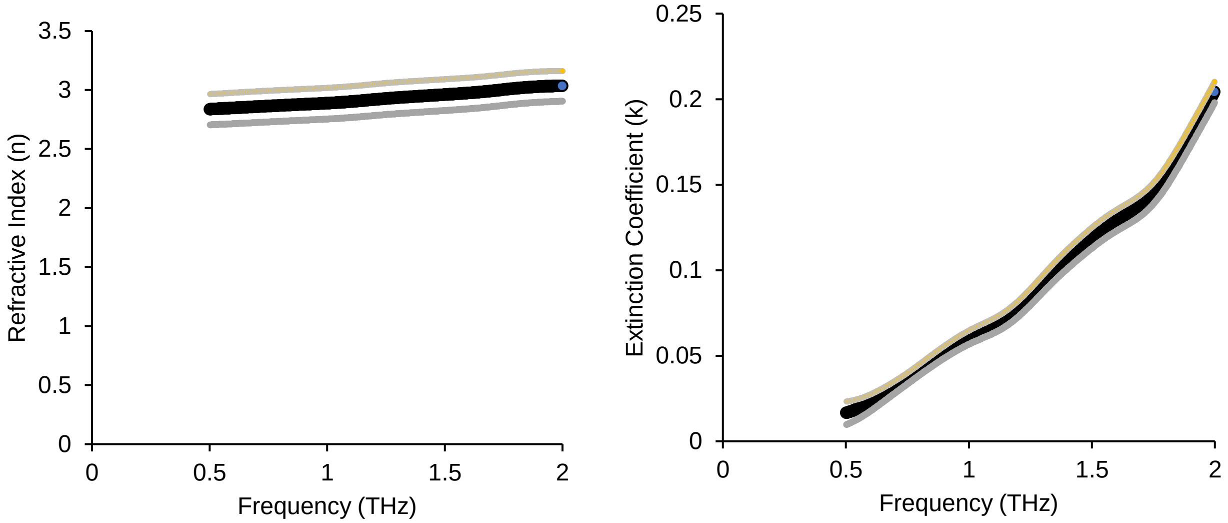

Suppl. Fig. S2. Optical properties (refractive index and extinction co-efficient) of broken eggshells with membrane at 0.5-2.0 THz region. (a) Refractive index of white eggshell with standard deviation. (b) Extinction co-efficient of white eggshell with standard deviation. The measurement was conducted using THz-TDS with transmission mode. The value of extinction co-efficient can differ based on the moisture content of the eggshell membrane or drying condition of the sample.

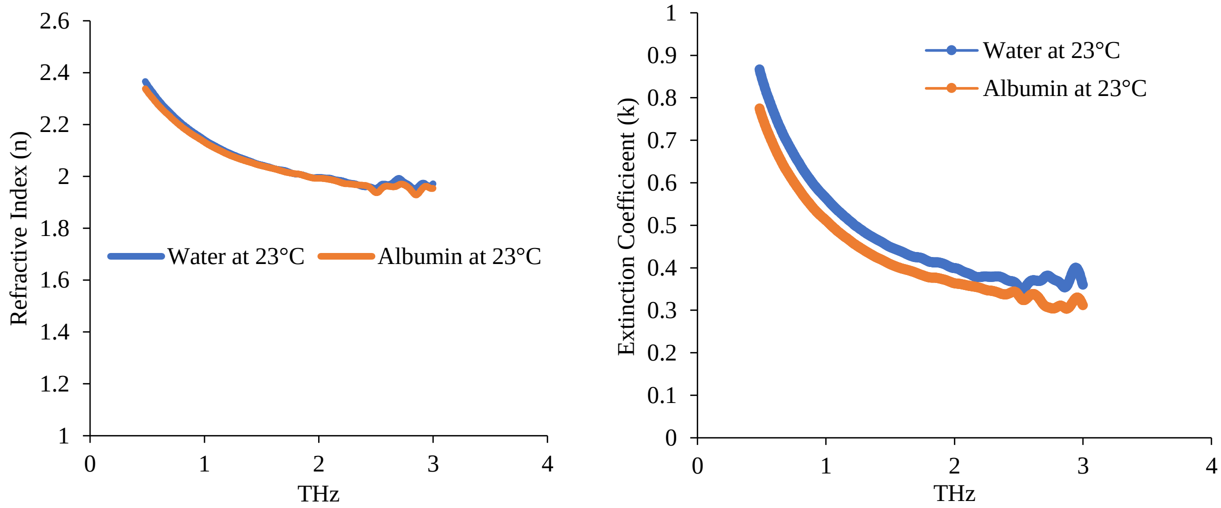

Suppl. Fig. S3. Optical properties (refractive index and extinction co-efficient) of egg white (i.e. albumen) at 0.5-3.0 THz region. (a) Refractive index of egg albumen. (b) Extinction co-efficient of egg albumen. The measurement was conducted using THz-TDS (ATR) method. ATR is the abbreviated form of attenuated total reflection.
